# Study on the Preferred Application-Oriented Index for Mental Fatigue Detection

**DOI:** 10.3390/ijerph15112555

**Published:** 2018-11-14

**Authors:** Tianhong Duan, Nong Zhang, Kaiway Li, Xuelin Hou, Jun Pei

**Affiliations:** 1 Key Laboratory of Deep Coal Resource Mining, School of Mines, Ministry of Education of China, China University of Mining and Technology, Xuzhou 221116, China; passionduan@163.com (T.D.); 18796281298@163.com (X.H.); ts18020191p21@cumt.edu.cn (J.P.); 2 State Key Laboratory of Coal Resources and Mine Safety, China University of Mining and Technology, Xuzhou 221116, China; 3 Department of Industrial Management, Chung Hua University, Hsinchu 30012, Taiwan; kai@chu.edu.tw

**Keywords:** mental fatigue, one-way ANOVA, digital decoding testing, relative fatigue index (RFI), sensitivity ordering

## Abstract

Most of the research on mental fatigue evaluation has mainly concentrated on some indexes that require sophisticated and large instruments that make the detection of mental fatigue cumbersome, time-consuming, and difficult to apply on a large scale. A quick and sensitive mental fatigue detection index is necessary so that mentally fatigued workers can be alerted in time and take corresponding countermeasures. However, to date, no studies have compared the sensitivity of common objective evaluation indexes. To solve these problems, this study recruited 56 human subjects. These subjects were evaluated using six fatigue indexes: the Stanford sleepiness scale, digital span, digital decoding, short-term memory, critical flicker fusion frequency (CFF), and speed perception deviation. The results of the fatigue tests before and after mental fatigue were compared, and a one-way analysis of variance (ANOVA) was performed on the speed perception deviation. The results indicated the significance of this index. Considering individual differences, the relative fatigue index (RFI) was proposed to compare the sensitivity of the indexes. The results showed that when the self-rated fatigue grade changed from non-fatigue to mild fatigue, the ranges of RFI values for digital span, digital decoding, short-term memory, and CFF were 0.175–0.258, 0.194–0.316, 0.068–0.139, and 0.055–0.075, respectively. Correspondingly, when the self-rated fatigue grade changed to severe fatigue, the ranges of RFI values for the above indexes were 0.415–0.577, 0.482–0.669, 0.329–0.396, and 0.114–0.218, respectively. These results suggest that the sensitivity of the digital decoding, digital span, short-term memory, and CFF decreased sequentially when the self-evaluated fatigue grade changed from no fatigue to mild or severe fatigue. The RFI individuality of the speed perception deviation is highly variable and is not suitable as an evaluation index. In mental fatigue testing, digital decoding testing can provide faster, more convenient, and more accurate results.

## 1. Introduction

With the development of modern industry and advancements in science and technology, the intensity and universality of mental work have gradually increased, which is the main inducement of mental fatigue. Mental fatigue is a state caused by long-term mental labor or the use of computers. It not only affects the efficiency of workers who undertake a lot of mentally taxing work, but also greatly affects their safety, health, and comfort. It is an interesting discovery that Hadi et al. [1] found that occupational safety and health can significantly improve the productivity of the staff of network maintenance sites. Mental fatigue is an important topic in occupational safety and health, and as the occupational safety and health problems of those undertaking mental work has begun to attract the attention of scholars, mental fatigue has gradually attracted increasing attention [2]. Yoshioka et al. and Uchino et al. [3,4] found that mental fatigue was very common in Japan. When the human body is in an exhausted fatigue state for an extended period of time, it may lead to harmful symptoms such as loss of appetite, nausea and vomiting, and even death from overwork [5,6]. According to a survey conducted in 2014, China has become the country with the most deaths due to overwork. More than 1600 people die every day due to over-fatigue. Additionally, many surveys have shown that the number of deaths from overwork is significantly higher for those performing mental work than those of physical workers [7]. Mental workers in the mining industry are more prone to suffering from mental over-fatigue because of the following reasons. First, these mental workers suffer from stress due to safety production and other reasons, which usually leads to mental fatigue. Second, these workers often work overtime, which can easily lead to mental fatigue. Third, undesirable underground working environments such as high temperature, high humidity, strong noise, high dust, low illumination, and so on will also aggravate mental fatigue. Last, most mines are in remote rural areas where medical, entertainment, sports, and other conditions are relatively poor. In addition, some workers who have been away from home for a long time can hardly get relief and recovery in time after mental fatigue. These mental workers in underground environments are more prone to accidents when they are suffering from mental fatigue.

A quick and sensitive mental fatigue detection index is necessary so that these kind of workers such as mental workers in the mining industry can be alerted to mental fatigue in time and take corresponding countermeasures. This is of great significance for reducing injuries caused by over-fatigue. At present, the evaluation methods for mental fatigue are divided into two categories: subjective testing and objective evaluation. The accuracy and convenience of these evaluations are quite different. Common mental fatigue subjective testing scales include the Stanford Sleepiness Scale [8] and the Swedish Occupational Fatigue Inventory [9,10]. Objective evaluation methods mainly evaluate the eye characteristics, facial features, brain waves, heart rate, and blood biochemical indexes of workers. Mental fatigue detection methods tend to combine multiple indexes for a comprehensive evaluation. Charbonnier et al. [11] divided the brain into six regions and combined the index of the alpha band of each brain region with the detection of ocular symptoms to propose a long-term mental fatigue detection method. However, each index needed to be detected every 20 s, and the detection process is complicated. Zhang and Yu [12] combined electroencephalogram (EEG) and heart rate variability (HRV) to identify different mental fatigue states. However, this method uses numerous instruments and equipment, and its large-scale application is difficult. Wang [13] conducted psychological and physiological measurements on pilots in different mental fatigue states to detect the fatigue state accurately using five simple methods: the NASA task load index (NASA-TLX) scale, heart rate variability, critical flicker fusion frequency (CFF), static posture, and reaction time. However, this method is also complex and time-consuming. The experimental process for EEG, eye movement, and other methods is in a similar state. There are some indexes which can be fast and convenient. However, no studies have compared the sensitivity of these indexes. Without study into this problem, it is impossible to propose a preferred index to evaluate mental fatigue.

In summary, there are still some deficiencies in the study of mental fatigue detection. First, most of the evaluation methods in studies on mental fatigue generally require instruments that are not easy to carry out as they are time-consuming and limited in application. Second, no studies have compared the sensitivity of the indexes.

To address these problems, an objective evaluation index with high sensitivity is preferred to promote the development of mental fatigue detection that is fast, convenient, and accurate. This study selected six methods for evaluation: the Stanford sleepiness scale, digital decoding, digital span, speed perception deviation, short-term memory, and CFF. This study verified the significance of the speed perception deviation index through a one-way analysis of variance (ANOVA). In addition to considering individual differences, the sensitivity of each index was compared using the relative fatigue index (RFI) to determine the sensitivity ranking of each index. Moreover, the detection time and convenience of each index were also compared. Based on the evaluation results, a preferred index was selected.

## 2. Experiments

### 2.1. Subjects

The subjects were all college students in Jiangsu Province, China. This study was conducted with 50 males and six females between 20 and 22 years old (mean 21.76 years; standard deviation 0.56). They were all juniors and were physically and mentally healthy, with no color blindness, color weakness, or any other symptoms; their visual acuity or corrected visual acuity and hearing were normal. They were required to avoid strenuous exercise, tobacco, alcohol, and other foods that may cause the central nervous system to be excited or suppressed for one day before the experiments. This study was conducted in accordance with the Declaration of Helsinki and was approved by the ethics committee of the China University of Mining and Technology (CUMT20170309SOM01). Written informed consent was obtained from every participant. All subjects understood the experimental process and volunteered to participate in the experiments.

### 2.2. Experimental Process

The experiments were conducted under two conditions. The first was under the condition of no fatigue. The subjects were required to be in an idle state for one day before testing, avoiding stimulating activities and ensuring they received over 8 h of sleep. Then, the first experiment was conducted the next day. The second experiment was conducted after fatigue. Each of the subjects had prepared for and participated in three examinations within two weeks before the second experiment. On the day of the experiment, they were deprived of a lunch break before the afternoon examination. Mental fatigue detection was performed immediately after the afternoon examination. The experiments consisted of two parts: subjective testing and objective evaluation. Subjective testing was performed using the Stanford sleepiness scale. The evaluation results included three grades: non-fatigue, mild fatigue, and severe fatigue. The objective tests consisted of five parts: digital span, digital decoding, speed perception deviation, shot-term memory, and CFF testing.

**Figure 1:**
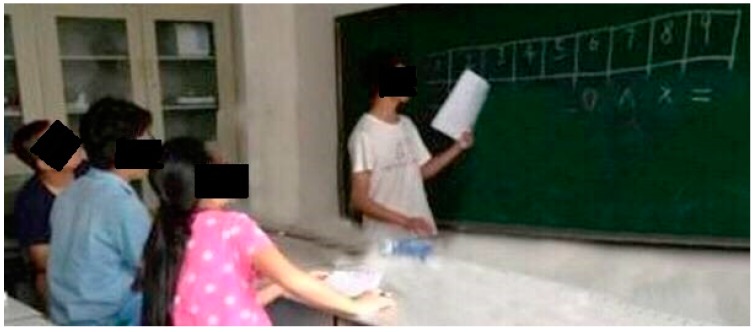
Experimental scene during the digital decoding testing.

For the digital span testing, an experimental assistant read 10-digit sequences consisting of numbers 1–9 to each subject, and each subject was required to repeat them at a specified time. Each experiment included nine sequences, and the subjects received one point per correct answer. During the digital decoding testing, the assistant read 14-digit sequences consisting of the numbers 1–9 to each subject. Each subject was then required to translate the sequences to the characters corresponding to each number according to the rules given in Table 1 within 90 s. No equipment was needed in these two experiments, and the assistants could be substituted by questionnaires in industrial applications, making these two methods very convenient in practice. The experimental scene during the digital decoding testing is shown in Figure 1.

**Figure 2:**
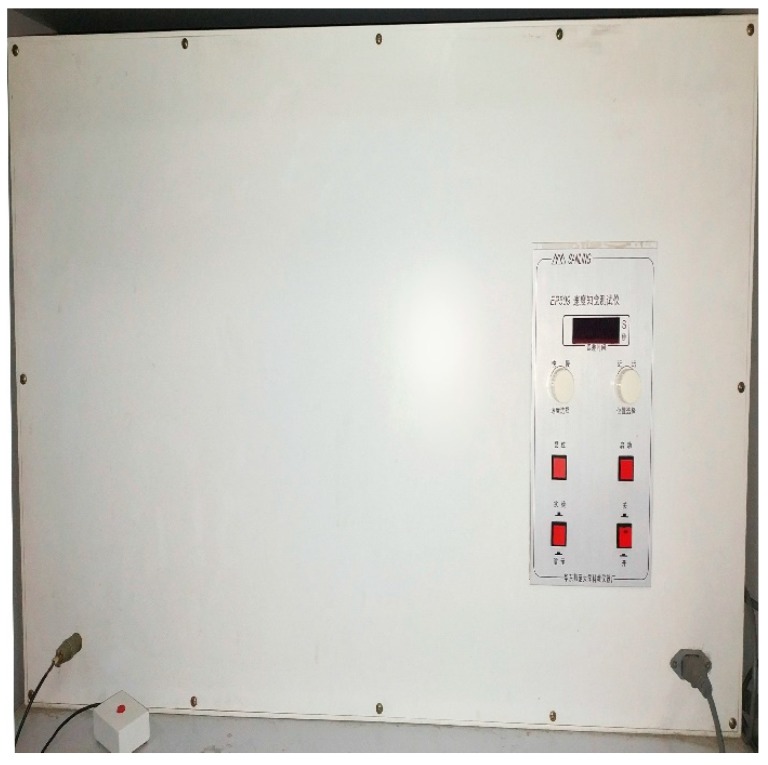
Speed perception deviation tester.

Speed perception deviation testing was completed by the main tester and the subject. First, the main tester adjusted the dial of the EP509 speed perception deviation tester (shown in Figure 2) to ‘slow’ + ‘far’, then pressed the light source switch. The light spot then moved from right to left, but was blocked when it entered a baffle. The subject needed to assume that the light spot was still moving behind the baffle at the original speed, and pressed a response key when they thought the light spot would reach the end. The error in the estimated time of the subject was displayed by the tester. As it needs two people in the experiment and the instrument is too large to carry around, this method is relatively less convenient in practical applications.

**Figure 3:**
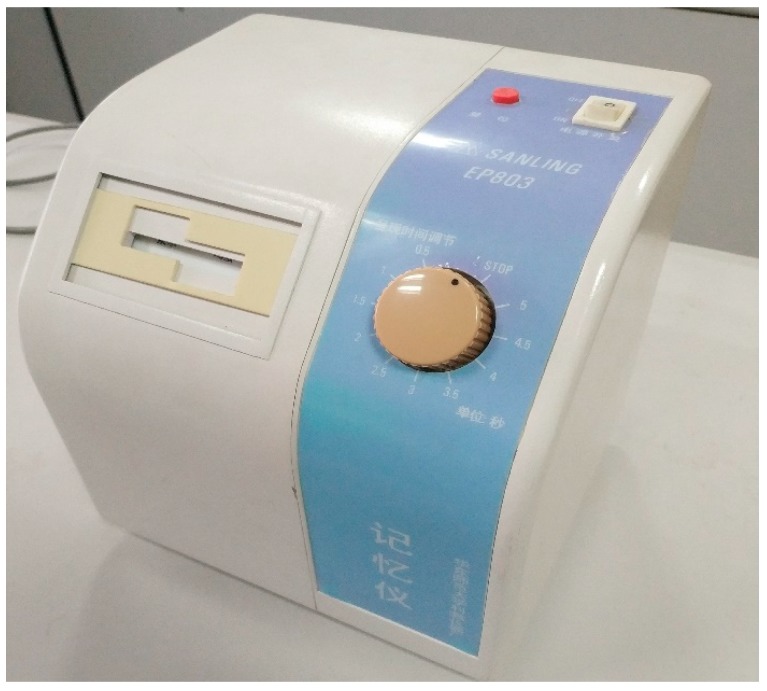
EP803 memory ability tester.

Short-term memory testing was also completed by two people using the EP803 memory ability tester (shown in Figure 3). First, the paper tape was played twice, and the subjects memorized the relationships between the words on the paper tape. At the beginning of the test, the main tester covered the word on the right side of the paper tape in the display box while the subject read the words on the left side of the tape and spoke the corresponding word on the right side before the tape was rotated to the next word. The main tester then judged and recorded the answers. As it needs two people and at least 150 s to complete one experiment for each subject and the words on the tape should be changed frequently, this method is not very practical in industrial applications.

**Figure 4:**
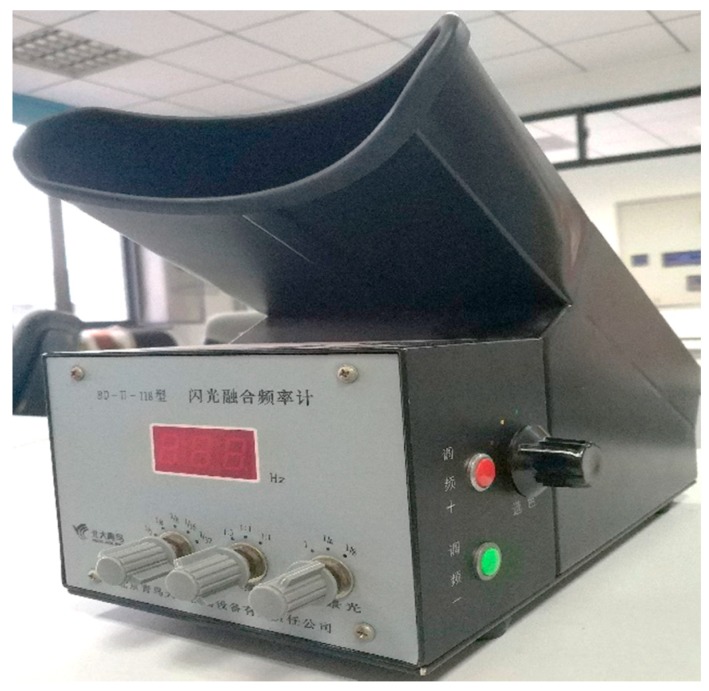
EP403 highlight scintillometer.

The CFF testing used an EP403 highlight scintillometer (shown in Figure 4). First, the flickering light was set as a ‘yellow’ color. During detection, the subject approached the lens hood and looked at the light source in the hood. The frequency of the light was gradually reduced from its highest value until the subject recognized that the light source started to flicker; the frequency of the light source at this time was recorded as the flicker frequency. Next, the frequency of the light was adjusted from its lowest level until the subject saw the light source stop flickering. The frequency of the light source at this time was recorded as the fusion frequency. Each test was repeated six times, and the average value of six flickers and six fusion frequencies was calculated to obtain the CFF value for the subject. This also requires two people and at least 180 s to complete one experiment for each subject; as a result, this method is also relatively impractical in industrial applications.

## 3. Experimental Results

### 3.1. General Laws

During subjective testing, subjects may deliberately hide their subjective feelings due to various motivations. However, in these experiments, all of the subjects were students, and the subjective testing results had no impact on the subjects. Thus, the subjects were not motivated to hide their subjective feelings. Moreover, the subjects were in a relaxed state and in a state of stressful mental fatigue before the first and second experiments, respectively. Therefore, the data obtained in these experiments can be considered reliable.

**Figure 5:**
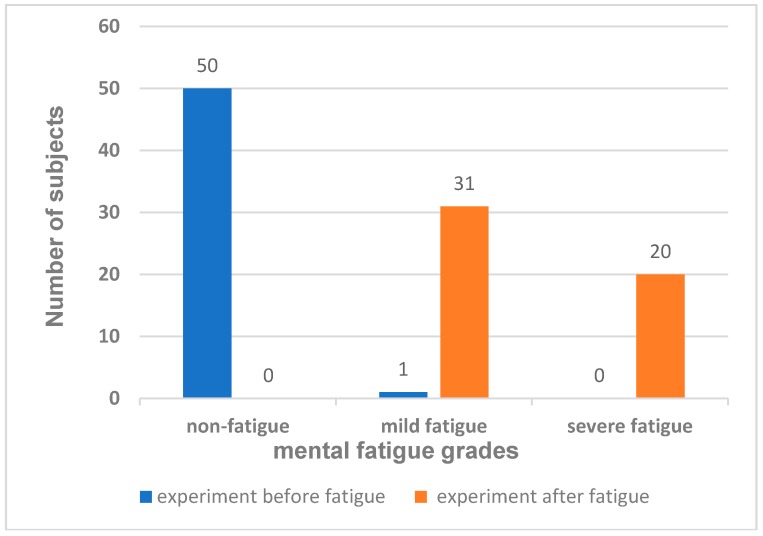
Number of subjects with each fatigue grade before and after mental fatigue.

This study analyzed the fatigue detection data for 56 subjects obtained before and after fatigue. The results of the subjective testing in the two experiments showed that 51 of the 56 subjects had a change in their self-rated fatigue grades before and after fatigue. To ensure the accuracy of the experimental results, data from the five subjects with no change were excluded as abnormal values. In the first experiment, 50 subjects had a non-fatigue grade, accounting for 98.0% of the total; one had a mild fatigue grade, accounting for 1.96%. In the second experiment, 31 subjects had a mild fatigue grade, accounting for 60.78%, while 20 subjects had a severe fatigue grade, accounting for 39.22%. Among them, a total of 31 formerly rated non-fatigue subjects later reported mild fatigue and 20 subjects later reported severe fatigue in mental fatigue grades, as shown in Figure 5.

The mean ($\bar{x}$) and standard deviation (σ) for all of the detection indexes of the three statuses (non-fatigue, mild-fatigue, and severe fatigue) are listed in Table 2. The experimental results showed a decrease in digital span, digital decoding, and short-term memory testing after mental fatigue. At the same time, in the CFF testing, the subjects were less sensitive in capturing the light flicker when they were in a fatigued state than in a relaxed state. The light source was generally considered to have stopped flickering at a lower flicker frequency when the subjects were in a fatigued state. In the speed perception deviation testing, the deviation of each subject generally increased. 

### 3.2. Significance Analysis of the Speed Perception Deviation Index

This study selected five mental fatigue detection indexes. Of these, digital decoding, digital span, short-term memory, and CFF testing have been validated in prior studies [13,14], but the effectiveness of the speed perception deviation index has not been validated. Therefore, this study used Minitab to analyze the speed perception deviation experiment data with a one-way ANOVA to evaluate the significance of this index.

In the one-way ANOVA, it is supposed that there are *r* levels in factor A, and the data in each level were independent. It is supposed that there were *n_i_* observed data in each level, and the number of total observed data was *n*. The degrees of freedom, *df*, corresponding to the three sum of squares calculations were determined to eliminate the influence of data variation on the sum of squares. The test statistic *F* was constructed, which is the ratio of mean squares for fact A (*MS_A_*) and mean squares for error (*MS_e_*). The F test was used to evaluate the significance of the data before and after fatigue.

The speed perception deviation testing data for the 31 subjects who changed from non-fatigue to mild fatigue states and 20 subjects who changed to severe fatigue states were analyzed under a significance level of α = 0.05. The results are summarized in Table 3. 

For the speed perception deviation testing, the variance analysis results for the subjects who changed from non-fatigue to mild fatigue states were F = 51.24 > F (1, 60) = 4 and *p* < 0.05. The variance analysis results for the subjects who changed to severe fatigue states were: F = 254.07 > F (1, 38) = 4.08 and *p* < 0.05. These results indicate that the values of the speed perception deviation index changed significantly after mental fatigue.

## 4. Proposal of A Relative Fatigue Index

**Figure 6:**
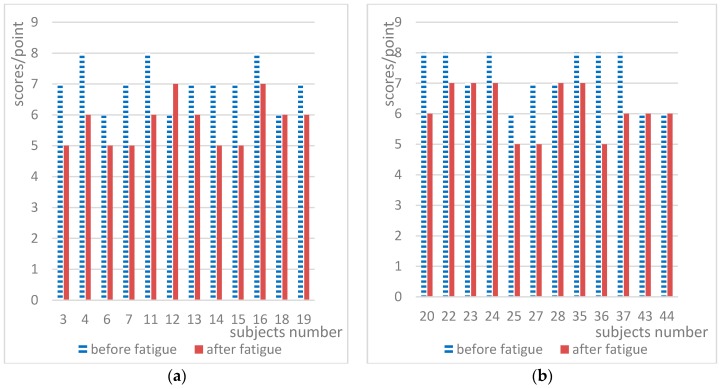

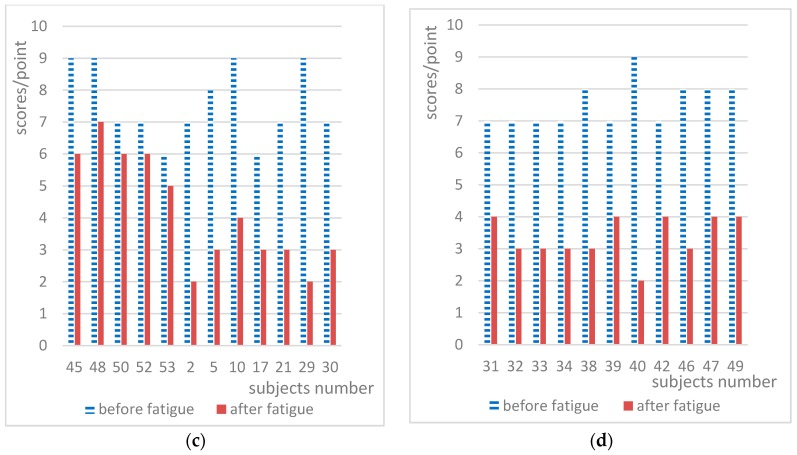
Digital span index scores for the two experiments.

**Figure 7:**
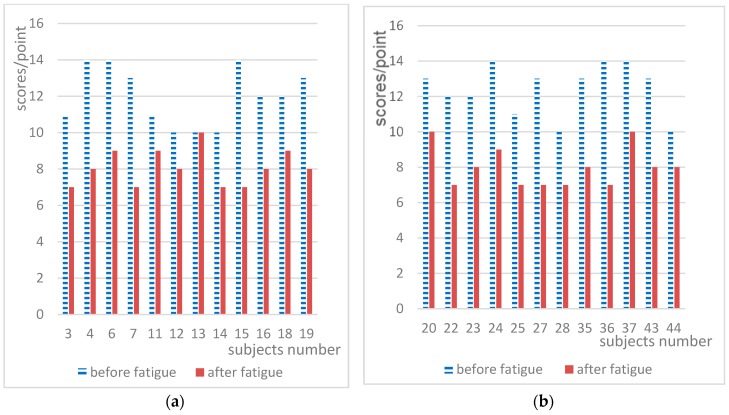

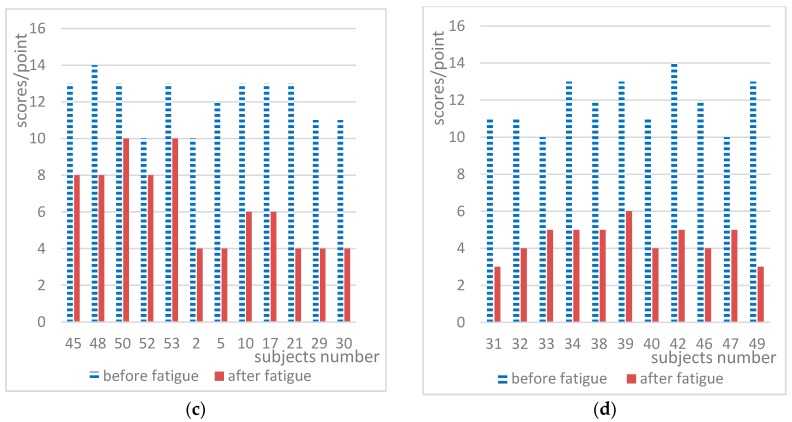
Digital decoding scores for the two experiments.

**Figure 8:**
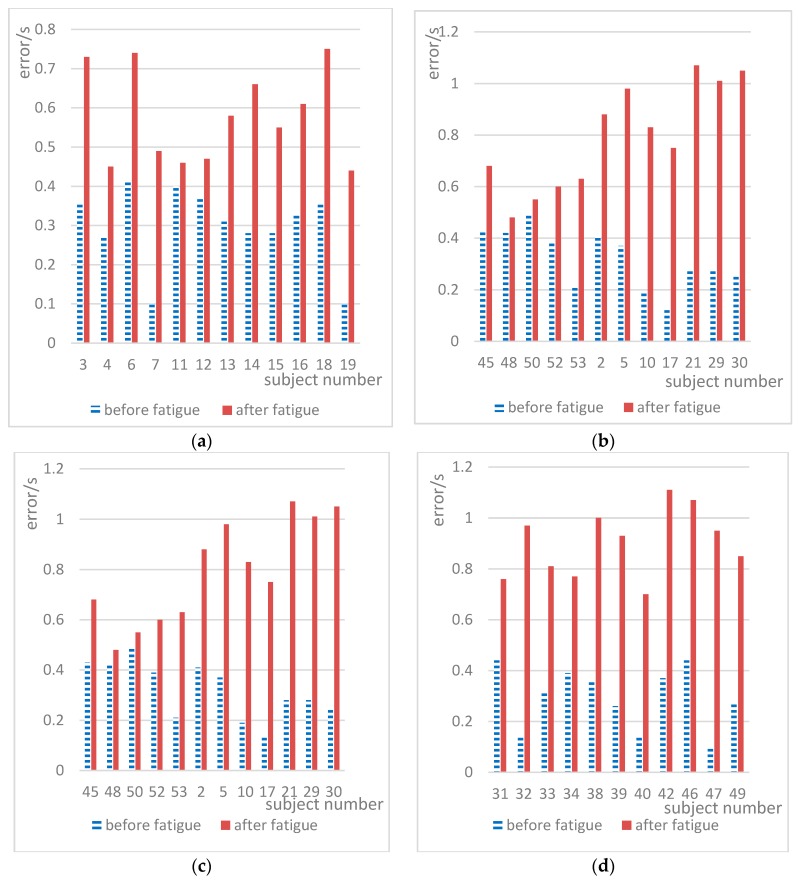
Speed perception deviation results for the two experiments.

It was found that there were very large differences among the data for subjects. There were 51 datapoints to analyze. However, the subjective self-rated fatigue grade of five subjects did not change, so these five subjects were excluded, and the data for the remaining 47 subjects was analyzed. The value of each objective index obtained from the two experiments before and after fatigue is shown as a histogram in Figure 6, Figure 7 and Figure 8, where the x-axis represents the subject number and the y-axis represents the testing values for each objective index. 

Figure 6 shows the digital span index scores for the experiments before and after fatigue. From Figure 6, it can be observed that the scores for all subjects were in the ranges of 6–9 and 2–7 before and after fatigue, respectively. Individual differences between the subjects were obvious, and there was an overlapping range of scores before and after fatigue. However, as can be seen from Figure 6, after mental fatigue, the digital span index scores of most subjects decreased. After fatigue, 40 subjects exhibited a decrease in the digital span index, accounting for 87.23%; one had an increase in the index, accounting for 2.13%. The number of subjects with unchanged scores was five, accounting for 10.64%. The scores of the majority of subjects dropped. 

Figure 7 shows the digital decoding scores of each subject obtained from the experiments before and after fatigue. In Figure 7, the scores of all subjects were in the ranges of 10–14 and 3–10 before and after fatigue, respectively. The score of a subject after fatigue could be the same as the score of another before fatigue, and the difference between individuals was obvious. However, as can be seen from Figure 7, the scores of the majority of subjects decreased after mental fatigue. After fatigue, 46 subjects exhibited a decrease in the digital decoding score, accounting for 97.87%; no one had abnormal scores, accounting for 0%; and the number of subjects with unchanged scores was one, accounting for 2.13%.

Huge differences in short-term memory and CFF between individuals were also found in the current study. The short-term memory and CFF scores for the majority of subjects decreased after mental fatigue. Figure 8 shows the speed perception deviations for the experiments before and after fatigue. In Figure 8, the scores of all subjects before were in the range of 0.10–0.49 and 0.44–1.11 before and after fatigue, respectively. The difference between individuals was obvious, and there was an overlapping range of scores before and after fatigue. However, as can be seen in Figure 8, the speed perception deviation for the majority of subjects increased after mental fatigue. After fatigue, 47 subjects exhibited an increase in the speed perception deviation, accounting for 100%. 

In summary, the huge differences between individuals indicate that it is unrealistic to use the absolute values of these indexes to evaluate individual fatigue. However, for most subjects, each index showed a consistent trend in change after mental fatigue. Likewise, we could not use absolute heart rate to evaluate physical labor intensity for individuals. However, RMR (relative metabolic rate) is widely used to evaluate the physical labor intensity for individuals. Similarly, it was also impossible for us to use absolute force to assess muscle fatigue. However, Danuta Roman-Liu, Tomasz Tokarski , and Radosaw Kowalewski proposed a new index-force change index (FCI) to assess muscle fatigue and demonstrated that this index differentiated the muscle fatigue of the upper limb depending on the external load [15]. Therefore, to eliminate the huge differences between individuals, this study defined a relative fatigue index (RFI). The RFI is defined as the ratio of the absolute value of the difference between the testing index values under fatigue and non-fatigue conditions to the testing index value under fatigue, which reflects the sensitivity of the testing index to mental fatigue. Assuming that the values of fatigue testing index A are *NF* and *F* before fatigue and after fatigue, respectively, the equation for calculating the fatigue index is as follows:

(1) $$\;RFI=\frac{\left|\;NF\;-\;F\right|}{NF}\;$$

The RFI value can be used as an index of mental fatigue for an individual. Thus, it can be used to classify the mental fatigue grade. The one-way ANOVA on RFI values for the five indexes of the 31 subjects who changed from non-fatigue to mild fatigue states and subjects who changed to severe fatigue states was performed under a significance level of α = 0.05. The results are summarized in Table 4. 

As shown in Table 4, although the *p* value of RFI for speed perception deviation was greater than 0.05, *p* values of RFI for the remaining four indexes were equal to 0.00. These results indicate that different degrees of mental fatigue have a significant effect on the RFI values of digital span, digital decoding, short-term memory, and CFF. This means that the developed indexes of RFI values for digital span, digital decoding, and short-term memory can be applied to the classification of mental fatigue grade.

## 5. Comparison of the Sensitivity of Each Index

The RFI value can be used to compare the sensitivities of the indexes. The RFI values for each testing index were calculated for each subject whose self-rated fatigue grade changed from non-fatigue to mild fatigue, and the ranges of the RFI values for each testing index are listed in Table 5.

**Figure 9:**
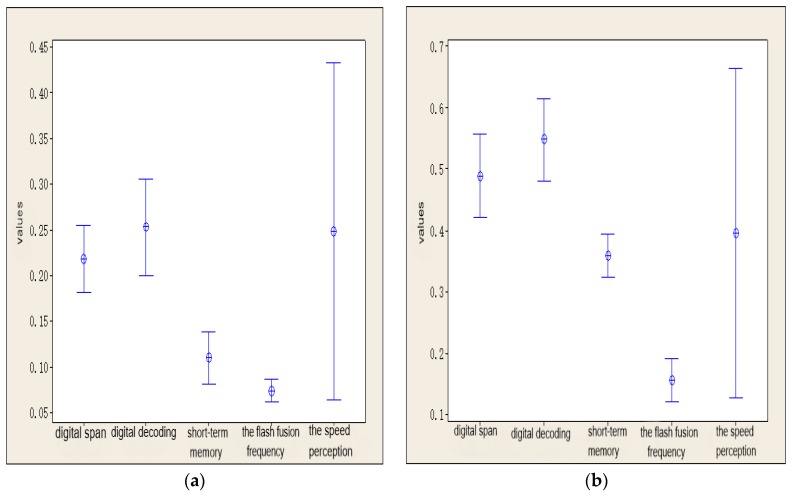
RFI values of different indexes. (**a**) From non-fatigue to mild fatigue; (**b**) From non-fatigue to severe fatigue.

In Figure 9a, the variation interval for each index for all subjects was drawn with a 95% confidence level. There were differences between the RFI values for each index of each subject. However, from the upper limit, lower limit, and mean value of each index, the RFI values (i.e., sensitivities) of the indexes could be sorted as follows: digital decoding > digital span > short-term memory > CFF; the individual differences in the RFI of the speed perception deviation value were quite large.

Similarly, the RFI values for each testing index were calculated for each subject whose self-rated fatigue grade changed to severe fatigue. The resulting RFI range for each testing index is listed in Table 6.

As can be seen from the data in Table 4 and Table 5, with an increase in the self-rated fatigue grade of the subjects, the RFI values of the indexes also increased correspondingly. In Figure 9b, the variation intervals of the indexes for all subjects were drawn with a 95% confidence level. There were differences between the RFI values of each index for all subjects. However, from the upper limit, lower limit, and mean value of each index, the RFI value (i.e., sensitivity) of the indexes could be ranked as follows: digital decoding > digital span > short-term memory > CFF. The individual differences in the RFI for the speed perception deviation value were again quite large. These two laws were the same as those for the subjects whose self-rated fatigue grades changed from non-fatigue to mild fatigue.

According to the above laws, regardless of the change in the fatigue state, the sensitivity of the detection indexes occurred in the same order: digital decoding > digital span > short-term memory > CFF > speed perception deviation. Therefore, in practical application, the RFI value for digital decoding is the preferred evaluation index for mental fatigue. However, the individual differences in the speed perception deviation were very large. Thus, it is not suitable as an evaluation index.

## 6. Discussion

Mental fatigue is a complex physiological and psychological phenomenon [16], which does not have a unified definition in academia [17]. At present, mental fatigue refers to a state of diminished alertness and overall performance decline caused by prolonged cognitive activity [18,19]. Work stress is the main cause of mental fatigue [20]. The state of human mental fatigue is difficult for others to identify, but workers have a certain perception of their fatigue state. In this study, the results of the Stanford sleepiness scale test showed that after a long period of work, subjects experienced mild or severe fatigue. After mental fatigue, there are a series of changes in the human body such as decreased decision speed [21], decreased selective attention [22,23], and changes in the EEG and heart rate [24]. In our objective mental fatigue testing, each subject generally showed a decrease in the correct answer rate in the digital span, digital decoding, and short-term memory tests after mental fatigue. Moreover, the subjects were less sensitive in capturing light source flickering, which was consistent with the results of Maedaet et al. [25]. In the speed perception deviation testing, the deviation in each subject’s judgment of the actual running speed of the light spot gradually increased after mental fatigue. 

Significance analysis of the speed perception deviation index indicates that the values changed significantly after mental fatigue. Most books or papers have merely discussed the indexes including digital span, digital decoding, short-term memory, and CFF used in this paper that change significantly after mental fatigue. However, as demonstrated in Figure 6, Figure 7 and Figure 8, the differences in these indexes before and after mental fatigue between individuals are obvious. Therefore, it is impossible to use the absolute values of these indexes to classify individual mental fatigue. Thus, we proposed a relative index, RFI, to solve the problem that the differences of the absolute indexes between individuals are obvious. RFI also provides a method to compare the sensibility of the indexes used in the experiments in this study on mental fatigue evaluation. This method leads to the conclusion of the sensitivity order of these indexes.

Our results showed that the sensitivity order of these testing indexes was the same when the self-rated fatigue grades changed from non-fatigue to either mild fatigue or severe fatigue. The individual differences in the results of the speed perception deviation testing were large. Speed perception deviation testing was used to measure the speed perception deviation of the subjects, and this deviation was an accumulation of the estimation deviation in the light spot arrival time and the brain interpretation and reaction delay. As a result, the deviation was relatively large, but the absolute values of the speed perception deviation testing were small, thus the range of the fatigue index corresponding to the speed perception deviation index was large. In contrast, the digital decoding testing had the highest sensitivity. In practice, there are both advantages and disadvantages of using digital span, digital decoding, CFF, and short-term memory testing, as summarized in Table 7. Taking all factors including the characteristic of convenience compared in the section of experiment process into account, digital decoding testing is the fastest, most convenient, and most effective method.

This study evaluated the indexes for each subject under states of non-fatigue and fatigue. However, considering individual differences, how to use RFI to specify the mental fatigue degrees of mental workers was not investigated. Moreover, this study was conducted among juniors in one university in China, so the applicability of the RFI index in other populations still has to be verified in future study. Furthermore, the relationship between mental fatigue and the factors that determine mental fatigue such as the intensity, duration, temporal demand, and frustration level of mental work was not investigated in this study. These will be the emphases of our future study.

## 7. Conclusions

It was found that the results of the one-way ANOVA of the speed perception deviation testing indicated that the speed perception deviation changed significantly after mental fatigue. The individual differences in digital decoding, digital span, short-term memory, and CFF were obvious; however, after mental fatigue, the change tendencies of each index were very consistent and exhibited little individual difference. The calculation results for the RFI showed that with an increase in the self-rated fatigue grade of the subjects, the RFI values of the indexes also increased correspondingly. The sensitivities of digital decoding, digital span, short-term memory, and CFF decreased sequentially. The individuality of the RFI for the speed perception deviation was highly variable and was not suitable as an evaluation index. Therefore, in practical applications, the digital decoding index may be preferred to provide detection that is faster, more convenient, and more accurate.

## Figures and Tables

**Table 1:** Digital symbol control table.

1	2	3	4	5	6	7	8	9
＿	⊥	コ	∟	∪	Ｏ	Λ	Ⅹ	＝

**Table 2:** Detection results for various fatigue indexes of the three statuses ($\bar{x}$ ± σ).

Status	Digital Span (Score)	Digital Decoding (Score)	Short-Term Memory (Score)	CFF (Hz)	Speed Perception Deviation (s)
Non-fatigue	7.20 ± 0.90	10.80 ± 2.0	25.72 ± 1.81	49.95 ± 4.29	0.44 ± 0.24
Mild fatigue	5.55 ± 0.93	7.77 ± 0.81	22.45 ± 1.71	45.74 ± 4.10	0.55 ± 0.36
Severe fatigue	3.65 ± 1.09	4.75 ± 1.33	16.80 ± 2.07	42.71 ± 3.78	0.62 ± 0.26

**Table 3:** Significance analysis of the speed perception deviation index.

Fatigue State Transition	Sources of Variation	SS (Sum of Squares)	df	MS (Mean of Squares)	F	*p* (Significance)
Non-fatigue to mild fatigue	Mental fatigue	0.98	1	0.98	51.24	<0.05
	error	0.60	60	0.01		
	Total	1.58	61			
Non-fatigue or mild fatigue to severe fatigue	Mental fatigue	3.58	1	3.58	254.07	<0.05
	error	0.48	38	0.02		
	Total	4.06	39			

**Table 4:** One-way ANOVA on RFI values for the five indexes.

Index	Sources of Variation	SS (Sum of Squares)	df	MS (Mean of Squares)	F	*p* (Significance)
RFI of Digital span	Mental fatigue	0.90	1	0.90	62.40	0.00
	error	0.70	49	0.01		
	Total	1.60	50			
RFI of Digital decoding	Mental fatigue	1.06	1	1.06	51.26	0.00
	error	1.02	49	0.02		
	Total	2.08	50			
RFI of Short-term memory	Mental fatigue	0.76	1	0.76	126.93	0.00
	error	0.29	49	0.01		
	Total	1.05	50			
RFI of CFF	Mental fatigue	0.08	1	0.08	28.73	0.00
	error	0.14	49	0.00		
	Total	0.22	50			
RFI of Speed perception deviation	Mental fatigue	0.26	1	0.26	0.93	0.34
	error	13.83	49	0.28		
	Total	14.09	50			

**Table 5:** RFI range for subjects who changed from non-fatigue to mild fatigue states.

Testing Index	RFI Range
Digital span	0.175–0.258
Digital decoding	0.194–0.316
Short-term memory	0.068–0.139
CFF	0.055–0.075
Speed perception deviation	0.055–0.440

**Table 6:** RFI range for subjects who changed to severe fatigue states.

Testing Index	RFI Range
Digital span	0.415–0.577
Digital decoding	0.482–0.669
Short-term memory	0.329–0.396
CFF	0.114–0.218
Speed perception deviation	0.122–0.675

**Table 7:** Comparison of the advantages and disadvantages of indexes.

Fatigue Testing Methods	Sensitivity Order		Testing Time (s)
	Non-Fatigue to Mild Fatigue	Non-Fatigue to Severe Fatigue	
Digital span	2	2	140
Digital decoding	1	1	120
Short-term memory	3	3	150
CFF	4	4	180
